# Supplementary material for: Mental Health Conditions– and Substance Use—Associated Emergency Department Visits during the COVID-19 Pandemic in Nevada, USA
Source: Int J Environ Res Public Health. 2023 Mar 1;20(5):4389. doi: 10.3390/ijerph20054389 (PMC10001596; doi:10.3390/ijerph20054389)
Supplement: Supplementary file 1 [file ijerph-20-04389-s001.zip › ijerph-2168144-supplementary.docx]

Table S1: ICD-10-CM codes for substance usage (opioids, cannabis, alcohol, and smoking), and mental health conditions (suicidal ideation, suicidal attempts, and schizophrenia)

|  | Descriptions | ICD-10-CM codes |
| --- | --- | --- |
| Substance uses (abuse, dependence, unspecified, poisoning) | Opioid | F11.10, F11.120, F11.121, F11.122, F11.129, F11.14, F11.150, F11.151, F11.159, F11.181, F11.182, F18.188, F11.19, F11.20, F11.220, F11.221, F11.222, F11.229, F11.23, F11.24, F11.250, F11.251, F11.259, F11.281, F11.282, F11.288, F11.29, 11.90, F11.920, F11.921, F11.922, F11.929, F11.93, F11.94, F11.950, F11.951, F11.959, F11.981, F11.982, F11.988, F11.99, T40.0X1, T40.0X2, T40.0X3, T40.0X4, T40.0X5, T40.0X6, T40.2X1, T40.2X2, T40.2X3, T40.2X4, T40.2X5, T40.2X6, T40.3X1, T40.3X2, T40.3X3, T40.3X4, T40.3X5, T40.3X6, T40.4X1, T40.4X2, T40.4X3, T40.4X4, T40.4X5, T40.4X6, T40.601, T40.602, T40.603, T40.604, T40.605, T40.606, T40.691, T40.692, T40.693, T40.694, T40.695, T40.696 |
|  | Cannabis | F12.10, F12.120, F12.121, F12.122, F12.129, F12.150, F12.151, F12.159, F12.180, F12.188, F12.19, F12.20, F12.220, F12.221, F12.222, F12.229, F12.250, F12.251, F12.259, F12.280, F12.288, F12.29, F12.90, F12.920, F12.921, F12.922, F12.929, F12.950, F12.951, F12.959, F12.980, F12.988, F12.99, T40.7X1, T40.7X2, T40.7X3, T40.7X4, T40.7X5, T40.7X6 |
|  | Alcohol | F10.10, F10.120, F10.121, F10,129, F10.14, F10.150, F10.151, F10.159, F10.180, F10.181, F10.182, F10.188, F10.19, F10.20, F10.220, F10.221, F10.229, F10.230, F10.231, F10.24, F10.25, F10.26, F10.27, F10.28, F10.29, F10.92, F10.94, F10.95, F10.96, F10.97, F10.98, F10.99, T51.0X1, T51.0X2, T51.0X3, T51.0X4, T51.1X1, T51.1X2, T51.1X3, T51.1X4, T51.2X1, T51.2X2, T51.2X3, T51.2X4, T51.3X1, T51.3X2, T51.3X3, T51.3X4, T51.8X1, T51.8X2, T51.8X3, T51.8X4, T51.91, T51.92, T51.93, T51.94 |
|  | Smoking | F17. 210 |
| Mental health conditions | Schizophrenia, other non-mood psychotic disorders | F20-F29 |
|  | Suicidal ideation | R45.851 |
|  | Suicidal attempt | X60-X84 |
